# Postoperative morbidity and mortality in pediatric indigenous populations: a scoping review and meta-analysis

**DOI:** 10.1007/s00383-023-05377-2

**Published:** 2023-02-16

**Authors:** Rachel J. Livergant, Georgia Fraulin, Kelsey Stefanyk, Catherine Binda, Sasha Maleki, Shahrzad Joharifard, Tracey Hillier, Emilie Joos

**Affiliations:** 1 Faculty of Medicine and Dentistry, University of Alberta, Edmonton, AB Canada; 2 Faculty of Medicine, University of British Columbia, Prince George, BC Canada; 3 Faculty of Medicine, University of British Columbia, Terrace, BC Canada; 4 Lower Mainland Pharmacy Services, Vancouver General Hospital, Faculty of Pharmaceutical Sciences, University of British Columbia, Vancouver, BC Canada; 5 Department of Pediatric and Thoracic Surgery, British Columbia Children’s Hospital, University of British Columbia, Vancouver, Canada; 6 Mi’kmaq Qalipu First Nation, Faculty of Medicine and Dentistry, University of Alberta, Edmonton, AB Canada; 7 Division of General Surgery, Trauma and Acute Care Surgery, Vancouver General Hospital, University of British Columbia, 767 West 12th Avenue, Vancouver, BC V5Z 1M9 Canada

**Keywords:** Indigenous, Surgical outcomes, Health equity

## Abstract

Mounting evidence suggests that childhood health is an important predictor of wellness as an adult. Indigenous peoples worldwide suffer worse health outcomes compared to settler populations. No study comprehensively evaluates surgical outcomes for Indigenous pediatric patients. This review evaluates inequities between Indigenous and non-Indigenous children globally for postoperative complications, morbidities, and mortality. Nine databases were searched for relevant subject headings including “pediatric”, “Indigenous”, “postoperative”, “complications”, and related terms. Main outcomes included postoperative complications, mortality, reoperations, and hospital readmission. A random-effects model was used for statistical analysis. The Newcastle Ottawa Scale was used for quality assessment. Fourteen studies were included in this review, and 12 met inclusion criteria for meta-analysis, representing 4793 Indigenous and 83,592 non-Indigenous patients. Indigenous pediatric patients had a greater than twofold overall (OR 2.0.6, 95% CI 1.23–3.46) and 30-day postoperative mortality (OR 2.23, 95% CI 1.23–4.05) than non-Indigenous populations. Surgical site infections (OR 1.05, 95% CI 0.73–1.50), reoperations (OR 0.75, 95% CI 0.51–1.11), and length of hospital stay (SMD = 0.55, 95% CI − 0.55–1.65) were similar between the two groups. There was a non-significant increase in hospital readmissions (OR 6.09, 95% CI 0.32–116.41, *p* = 0.23) and overall morbidity (OR 1.13, 95% CI 0.91–1.40) for Indigenous children. Indigenous children worldwide experience increased postoperative mortality. It is necessary to collaborate with Indigenous communities to promote solutions for more equitable and culturally appropriate pediatric surgical care.

**Supplementary Information:**

The online version contains supplementary material available at 10.1007/s00383-023-05377-2.

## Introduction

Safe and appropriate surgical care is an integral component of an effective and resilient healthcare system [1]. Surgical care is a growing need globally, with surgical conditions accounting for over 33% of the global burden of disease [2]. Substantial health inequities continue to negatively impact the health of Indigenous populations worldwide, notably in the Americas and Oceania [3, 4, 5, 6]. These inequities are driven by healthcare systems, practitioner factors and by socioeconomic and connectivity deficits related to colonization, globalization, loss of culture, racism, and disconnection from their traditional land [3, 7]. Minority pediatric populations face unique challenges due to both their age and vulnerability and, consequently, Indigenous children and adolescents living in settler-governed countries face some of the largest health inequities worldwide [8]. The consequences of racism on Indigenous children within healthcare institutions have been compared to the impacts of adverse childhood experiences (ACEs) such as abuse or neglect. Both have lifelong negative impacts on a child’s mental and physical health [8, 9]. On an international stage, pediatric Indigenous health has been recognized by calls from the United Nations Declaration on the Rights of Indigenous Peoples and Canada’s 2015 Truth and Reconciliation Commission for institutions to address health inequalities at the familial level [10, 11, 12].

Current data on postoperative outcomes in pediatric Indigenous patients remain limited and of poor quality [13]. Mapping the evidence that exists in the published literature regarding barriers to adequate surgical quality of care for pediatric Indigenous populations is necessary to understand the extent of the inequities in postoperative outcomes that exist worldwide. This scoping review aims to assess if and which inequities exist between surgical outcomes in pediatric Indigenous and non-Indigenous peoples on the American and Oceanic continents by comprehensively reviewing the existing literature and meta-analyzing the results.

## Methods

This scoping review and meta-analysis portion was registered in Open Science Framework (osf.io/qs3vz) and reported in accordance with Preferred Reporting Items for Systematic Review and Meta-Analysis-Extension for Scoping Reviews (PRISMA-ScR) and Meta-Analysis of Observational Studies in Epidemiology (MOOSE) guidelines (see supplementary data) [14, 15].

### Data sources and searches

A search strategy was developed in conjunction with a professional librarian. Comprehensive electronic database searches were undertaken in MEDLINE, Embase, Global Health, Cochrane Library, PsycInfo, SOCIndex, Web of Science, and ProQuest Dissertations & Theses Global from inception to December 25, 2022, using key MeSH terms. No restriction was placed on language. Complete search strategies of databases can be found in supplementary documents. Grey literature, reference lists of reviews and retrieved articles, and consultations with experts were also conducted to identify additional relevant studies.

### Study selection and criteria

Two reviewers independently screened titles, abstracts, and full texts (R.L. and G.F., C.B., K.S., or S.M.). Discrepancies were resolved via group consensus. Studies were included if they were experimental or observational studies and excluded if they were book chapters, conference abstracts, or non-peer reviewed articles. Regional differences exist with regards to the definition of “pediatric patients”. For the purpose of this study, multiple definitions of “pediatric” were included, as defined by the specific study. Studies were excluded if they focused on Indigenous populations outside of the Americas or Oceania, if they lacked a non-Indigenous comparator group, or if they included adult patients. Geography was restricted to these continents as they share similar European colonial settler histories and consequent displacement and oppression of native peoples to those lands different than those in Asian, African, European, and Middle Eastern Indigenous groups [16]. Studies describing minor interventions and procedures conducted by interventional radiologist, pulmonologists, gastroenterologists, hematologists, or interventional cardiologists, including angiography, bronchoscopy, colonoscopy, gastroscopy, bone marrow biopsies, and percutaneous procedures were excluded. If studies only described pre-operative or intraoperative outcomes, they were excluded.

### Data extraction and quality assessment

One reviewer (R.L.) completed data extraction and quality assessment (QA), while another reviewer (K.S., C.B., or S.M.) verified the extracted data and QA findings. The following data were extracted from included studies using Microsoft Excel (Microsoft Corporation, Version 16.60): authors’ name, journal, year of publication, age category, population sizes, sex, type of study, surgery specialty and operations performed, outcomes of interest, and study conclusions. Studies were included in data extraction if they reported the surgical procedure performed and at least one outcome of interest resulting from the procedure. Studies reporting on two separate Indigenous groups had data extracted independently for each unique group. Quality of studies and risk-of-bias assessment was conducted using the Newcastle–Ottawa Scale (NOS), adapted for observational studies [17]. To assess the risk of publication bias, the effect odds ratio (OR) for each of the included studies was plotted against their standard error on a logarithmic scale to produce a funnel plot. Funnel plots were assessed for asymmetry to indicate possibility of publication bias. Disagreements between reviewers regarding data extraction and QA ratings were resolved through consensus.

### Data analysis

A random-effects model was used to define all pooled outcome measures and the OR was estimated with its variance and 95% confidence interval (CI). The prevailing heterogeneity between ORs for the comparable outcomes between different studies was calculated using the I-squared inconsistency test that depicts the percentage of total variation across studies and reflects heterogeneity rather than chance. The absence of statistical heterogeneity was indicated by a value of 0%, whereas larger values indicate increasing heterogeneity. Studies were only eligible for inclusion in meta-analysis if data were reported which summary associations (ORs or RRs) and their 95% CIs could be calculated or these summary associations were provided in the study itself. All meta-analyses were carried out using Review Manager, Version 5.4 (Cochrane Collaboration, 2020).

Outcomes from studies were separated into categories of postoperative morbidity, postoperative mortality, or increased health system interactions. Morbidity included surgical infections (superficial and deep surgical site infections, anastomotic dehiscence), hematologic (postoperative anemia, hematoma, hemorrhage), pulmonary (pneumonia, aspiration), and immunologic (graft rejection, graft failure) postoperative complications. Mortality was divided into two categories: (1) in-hospital and 30-day mortality and (2) greater than 30-day mortality, which included overall mortality and survival. Increased health system interactions included readmission, reoperation, and length of hospital stay. Subgroup analyses were conducted based on surgical speciality, surgery performed, and geographic location. Sensitivity analysis compared fixed-effects to random-effects models to test the assumption that the random-effects method was the most appropriate choice for the analysis.

## Results

### Study selection and characteristics

**Fig. 1:**
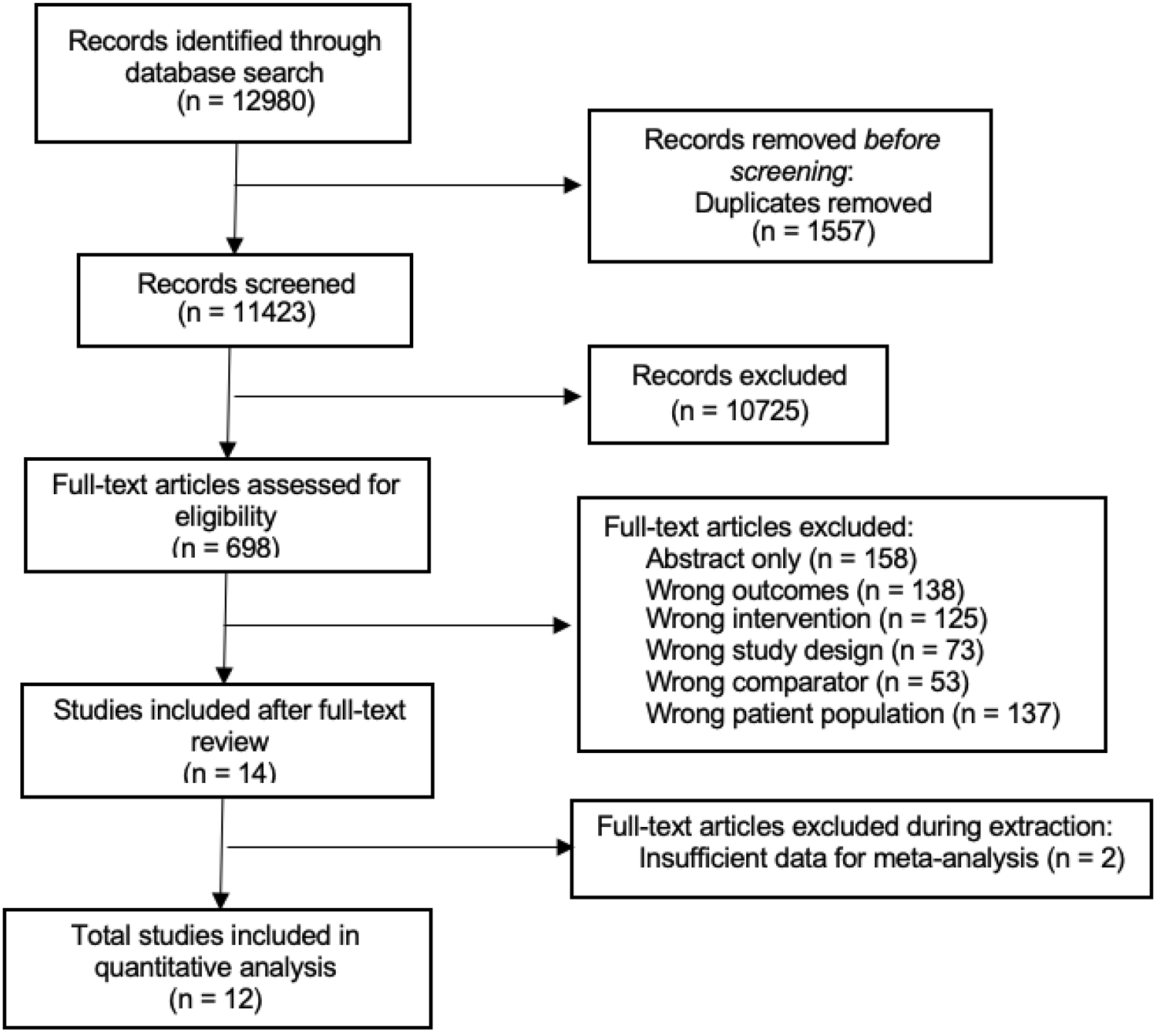
PRISMA flow diagram of study selection process, inclusions, and exclusions

A PRISMA flow diagram outlining the scoping review process is presented in Fig. 1. The initial search resulted in a total of 11,423 non-duplicate studies, of which 698 were included in full-text review after title and abstract review. Following full-text review and gray literature search, 14 unique studies met inclusion criteria, of which 12 were included in the final meta-analysis.

Of the 14 studies that met our inclusion criteria, all were retrospective studies, eight of which (57.1%) were retrospective cross-sectional cohort studies. A comprehensive summary of findings and characteristics of all included studies are presented in Table 1. The studies were published between 2009 and 2021, with research conducted from 1985 to 2016. A total of seven studies were based in Oceania (four New Zealand, three Australia), seven in NA (four USA, three Canada), and none were based in SA. Surgical outcomes were reported for 147,861 patients across six pediatric surgical specialties, including general surgery (*n* = 4), urology (*n* = 2), neurosurgery (NS) (*n* = 2), ear nose and throat (ENT) (*n* = 2), cardiac (*n* = 2), and ophthalmology (*n* = 1).

**Table 1:** Included study descriptions and summary of findings

First author, year	Study design; cohort years	Age group; participants* (*n*)	Surgical discipline; procedure(s)	M&M postoperative outcome category	Overall difference in M&M for IN	Overall quality assessment
North America						
Canada						
Beres 2010 [18]	Retrospective cohort; 2006–2008	Pediatric (< 21); total *n* = 210; Northern Aboriginal *n* = 68; local *n* = 142	Pediatric general surgery; appendectomy	Surgical site infections (wound infection/abscess); other morbidity: (perforation, ileus); postoperative readmission; LOS differences	No difference	Poor
Matsuda-Abedini 2009 [19]	Retrospective cohort; 1985–2005	Pediatric (< 18); total *n* = 159; Aboriginal *n* = 24; non-Aboriginal *n* = 135	Pediatric urology; renal transplantation	Immunologic complications (delayed-graft function, acute rejection, late rejection, eGFR at 5 years); > 30-day mortality (all-cause mortality)	Increased	Poor
Samuel 2011 [20]	Retrospective cross-sectional cohort; 1992–2007	Pediatric (< 22); total *n* = 291; Aboriginal *n* = 35; White *n* = 256	Pediatric urology; renal transplantation	Immunologic complications (first graft failure within 1-year)	Increased	Poor
USA						
Attenello 2015 [21]	Retrospective cross-sectional; 2000, 2003, 2006, 2009	Pediatric (< 21); total *n* = 16,941; Native American *n* = 155; white *n* = 16,786	Pediatric neurosurgery; ventriculoatrial shunt, ventriculopleural shunt, ventriculoperitoneal shunt, replacement of ventricular shunt	30-day mortality (30-day mortality, inpatient death); non-routine discharge	Increased	Good
Sherrod 2016 [22]	Retrospective cross-sectional cohort; 2012–2013	Pediatric (< 18); total *n* = 7228; Native American *n* = 57; Pacific Islander *n* = 24; White *n* = 7204	Pediatric neurosurgery; myelomeningocele repairs, spine procedures, craniectomy/craniotomy, shunt/ventricular catheter including revision/removal, skin lesions, other	Postoperative readmission (unplanned readmission)	Increased	Poor
Stone 2013 [23]	Retrospective cross-sectional; 2003, 2006	Pediatric (< 21); total *n* = 59,185; Native American *n* = 474; White *n* = 58,711	Pediatric general surgery; appendectomy, pyloromyotomy, intussusception, decortication, congenital diaphragmatic hernia repair, colonic resection for Hirschsprung's disease	30-day mortality (postoperative); Overall Morbidity (postoperative morbidity (wound (mechanical), infection, urinary, pulmonary, gastrointestinal, cardiovascular, systemic, and procedural); LOS	No difference	Good
Zwintscher, 2014 [24]	Retrospective cross-sectional cohort; 2009	Pediatrics (< 20); total *n* = 45,043; Native American *n* = 828; Caucasian *n* = 44,215	Pediatric general surgery; appendectomy	Surgical site infections (wound, dehiscence, gangrene; hematologic complications (anemia, hemorrhage, hematoma); pulmonary complications (pneumonia, other respiratory); postoperative reoperation (including for incisional hernias and postoperative obstruction) other postoperative complications (foreign body, UTI, shock)	No difference	Poor
Oceania						
Australia						
Chinnaratha 2014 [25]	Retrospective cross-sectional cohort; 1985–2012	Pediatric (< 16): total = 638; indigenous (Aboriginal Australian and Torres Strait Islander) *n* = 14; non-indigenous *n* = 622	Pediatric general surgery; liver transplantation	Overall morbidity: (1, 3, 5, 10, 15-year graft survival) > 30 day mortality: (1, 3, 5, 10, 15-year overall survival)	No difference	Poor
Jassar 2009 [26]	Retrospective cohort; 1996–2004	Pediatric (< 10); total *n* = 213; indigenous *n* = 111; non-indigenous *n* = 102	Pediatric ENT; tympanostomy	Surgical site infections (postoperative infection)	No difference	Poor
Justo 2017 [27]	Retrospective cohort; 2008–2014	Pediatric; total *n* = 1528; indigenous (Aboriginal Australian and Torres Strait Islander) *n* = 123; non-indigenous *n* = 1405	Pediatric cardiac surgery; CHD surgery	30-day mortality (30-day mortality, surgical survival 5 year unadjusted); LOS differences (ICU stay (days), hospital stay (days), surgical re-intervention)	Increased	Good
New Zealand						
Campbell 2016 [28]	Retrospective cohort; 2004–2013	Pediatric (< 15); total *n* = 153; Māori *n* = 34; European *n* = 119	Pediatric general surgery; cholecystectomy	Overall morbidity: operative complications (bile leak, bowel injury, bowel obstruction, cholangitis, wound infection, hemorrhage, cardiorespiratory, pseudocyst)	Increased	Poor
Chong 2021 [29]	Retrospective cross-sectional cohort; 2005–2014	Pediatrics (< 20); total *n* = 3414; Māori *n* = 698; PI *n* = 219; European *n* = 3195	Pediatric ophthalmology; strabismus repairs	Postoperative reoperation (reoperation rates for surgical failures)	Increased	Poor
Cloete 2019 [30]	Retrospective cohort; 2006–2014	Pediatric (< 18); total *n* = 144; Māori *n* = 38; PI *n* = 13; European *n* = 113	Pediatric cardiac surgery; HLHS, Aortic coarctation, interrupted aortic arch, aortic valve/supravalvular anomalies repairs	30-day mortality (postoperative mortality)	Increased	Poor
Johnston 2018 [31]	Retrospective cross-sectional cohort; 1996–2006	Pediatric (< 10); total *n* = 11,941; Māori *n* = 2387; non-Māori *n* = 9554	Pediatric ENT; Myringotomy + tube insertion	Postoperative Readmission (30-day readmission rate with surgical complication); postoperative reoperation; LOS differences. total number of readmissions for surgery	No difference	Poor

*CHD* congenital heart disease, *eGFR* estimated glomerular filtration rate, *ENT* ears, nose, throat, *HLHS* hypoplastic left heart syndrome, *ICU* intensive care unit, *IN* indigenous, *LOS* length of (hospital) stay, M&M mortality and morbidity, *PI* Pacific Islander

*Participant populations are listed as reported in individual studies

The studies evaluated outcomes for numerous surgical procedures, the most common ones being appendectomy (*n* = 104,438 participants in three studies); NS procedures including shunt placement (ventricular, ventriculoatrial, ventriculopleural, and ventriculoperitoneal), myelomeningocele repair, craniectomy, craniotomy, spinal procedures, skin lesion procedures, and other neurosurgical operations (*n* = 24,169 participants in two studies); tympanostomy and myringotomy with tube insertion (*n* = 12,154 participants in two studies); renal transplantation (*n* = 450 participants in three studies); strabismus surgery (*n* = 3414 participants in one study); cardiac surgeries including surgery for congenital heart defects (CHD), acquired heart disease, hypoplastic left heart syndrome (HLHS), aortic coarctation, interrupted aortic arch, aortic valve/supravalvular anomalies repairs, and others (*n* = 1672 participants in two studies); liver transplantation (*n* = 638 participants in one study); and cholecystectomy (*n* = 149 participants in one study). Study population-specific sample sizes ranged from 13 (Pacific Islander patients receiving cardiac surgery) to 44,215 (non-Indigenous comparator population receiving appendectomies).

A total of 88,385 patients were included in the meta-analysis; 4793 (5.4%) were Indigenous and 83,592 (94.6%) were non-Indigenous. Indigenous populations consisted of 3157 (65.9%) Māori, 1040 (21.7%) Native American, 248 (5.2%) Aboriginal Australians and Torres Strait Islander, 256 (5.3%) Pacific Islander Peoples, and 92 (1.9%) Indigenous Canadians.

Overall postoperative morbidity and mortality for Indigenous children in comparison to non-Indigenous children was increased in 8/14 studies (57.1%) and there was no significant difference in 6/14 studies. A decrease in morbidity and mortality for Indigenous populations in comparison to non-Indigenous patients was not reported in any studies within this review.

### Risk-of-bias assessment

**Fig. 2:**
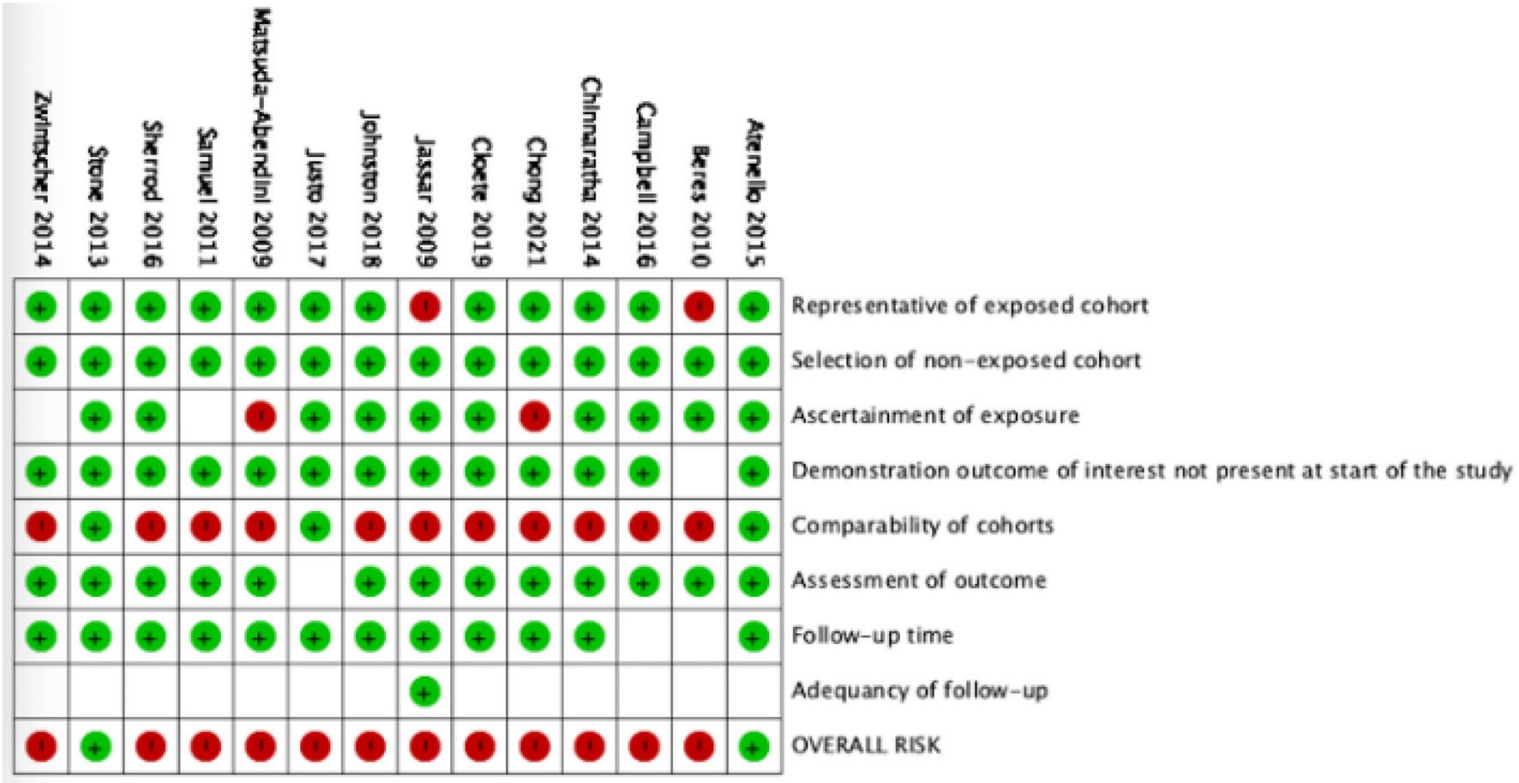
Risk of bias and quality assessment of included studies using the Newcastle Ottawa Scale. Red: High risk/low quality; white: unclear risk/fair quality study; green: low risk/good quality study

The majority of studies (*n* = 12, 85.7%) were high risk of bias and low quality, while two (14.3%) studies were low risk of bias and good quality [21, 23]. The low quality of studies was mainly attributed to failure of studies to control for significant differences, such as age, pre-existing co-morbidities, and/or sex in their analysis between Indigenous and non-Indigenous groups (*n* = 11, Fig. 2). Funnel plots for each outcome were generated; however, due to the inherent heterogeneity of the study and the low overall number of events for each outcome, asymmetry could not be reliably assessed.

### Postoperative morbidity

**Fig. 3:**
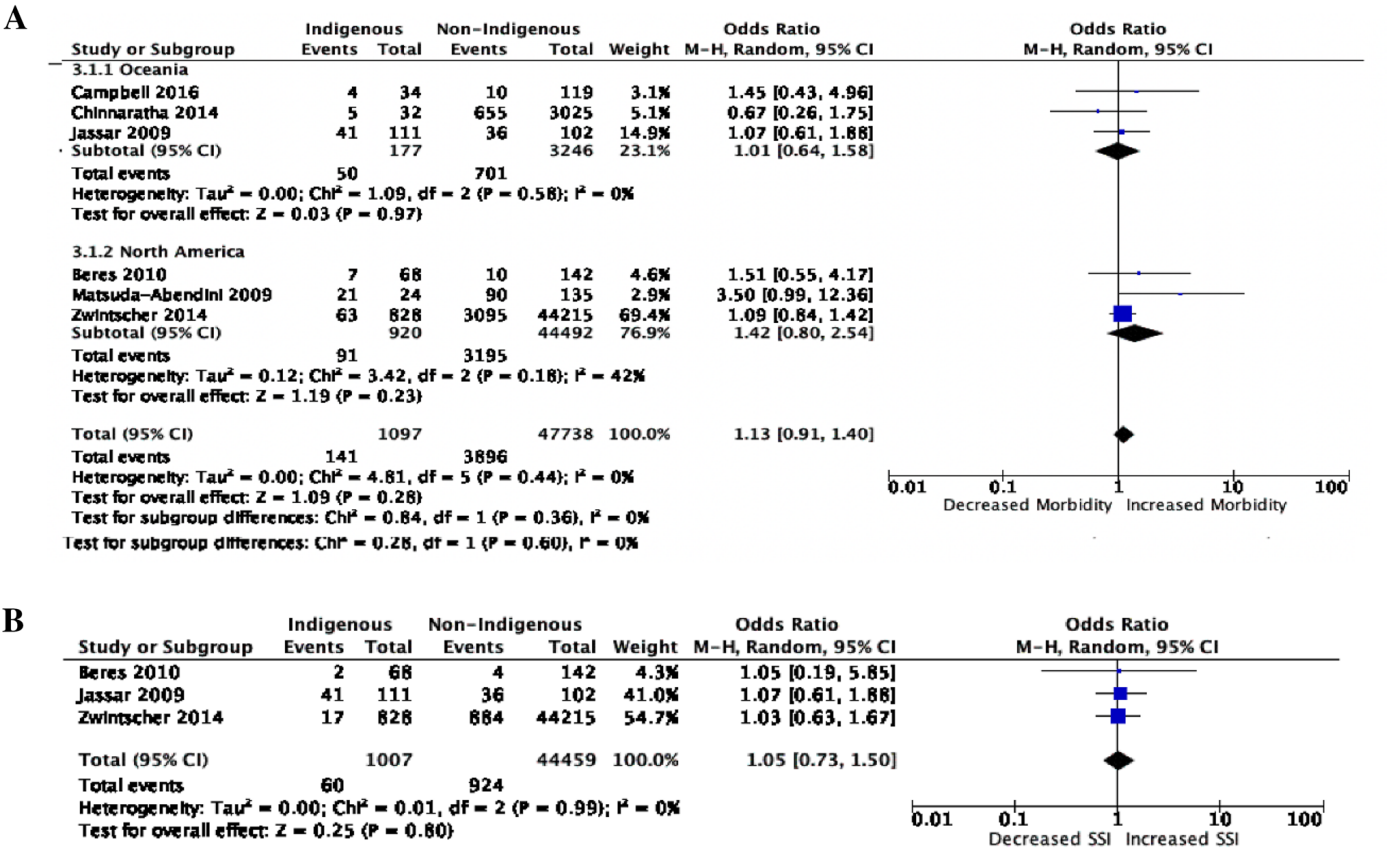
**A** Overall postoperative morbidity with sub-group analysis by geographic location and **B** postoperative surgical site infection (SSI) rate differences between Indigenous and non-Indigenous pediatric patients. Overall morbidity includes any postoperative complications, such as infectious, cardiac, respiratory, thromboembolic, bleeding, and immunologic complications

Seven studies addressed postoperative morbidity, of which six studies were included in the morbidity meta-analysis, representing 141 Indigenous patients and 3,896 non-Indigenous patients with postoperative complications [18, 19, 24, 25, 26, 28]. Overall, no significant difference was observed in postoperative morbidity between Indigenous and non-Indigenous pediatric patients (OR 1.13, 95% CI 0.91–1.40, *p* = 0.28; Fig. 3A). Studies were homogeneous in nature with an I-squared of 0%. When analyzed by geographic location, overall morbidity between Indigenous and non-Indigenous patients remained non-significant in both Oceania (OR 1.01, CI 0.64–1.58, *p* = 0.64; Fig. 3A) and North America (OR 1.42, CI 0.80–2.54, *p* = 0.23; Fig. 3A).

#### Surgical site infections

Three studies described postoperative SSIs including wound infections, wound dehiscence, abscesses, sepsis, and necrotizing infections [18, 24, 26]. A total of 60 Indigenous and 924 non-Indigenous children were included in this analysis who experienced postoperative surgical infections. There was no significant difference between the two groups with regards to the odds of experiencing a postoperative surgical infection (OR 1.05, 95% CI 0.73–1.50, *p* = 0.80; Fig. 3B).

#### Hematologic complications

One study provided data on hematologic postoperative complications, including hematomas, hemorrhages, and/or anemia from blood loss [24]. The study found that 0.8% (*n* = 7) of Native American children versus 0.5% (*n* = 221) of non-Indigenous children undergoing appendectomies experienced hematologic postoperative complications. However, no statistics on differences between the groups were presented in this study.

#### Pulmonary complications

One study provided data on postoperative pulmonary complications [24]. These complications included pneumonia and other, unspecified respiratory complications (including pneumothorax, respiratory distress, and/or aspiration). In this study, 1.4% (*n* = 12) of Native American children experienced pulmonary complications, compared to only 0.7% (*n* = 12) of non-Indigenous patients following appendectomies. Similar to above, no statistics on differences between the groups were presented in this study.

#### Immunologic complications

Three studies provided data on postoperative graft function and rejection after solid organ transplantation [19, 20, 25]. One of the studies did not find a significant difference in short-term outcomes post-transplantation including delayed-graft function and acute rejection between Indigenous and non-Indigenous children in Canada [19]. However, Indigenous children were found to have significantly poorer long-term graft survival than non-Indigenous children [19]. In the other study that examined immunologic complications, Indigenous children had a three times higher risk of renal graft failure compared to non-Indigenous children in Canada (HR 3.26, CI 1.51–7.03) [20]. The one study examining post-liver transplant graft survival in Aboriginal Australians and Torres Strait Islander children did not find a significant difference in graft survival between the Indigenous and non-Indigenous Australian children [25].

### Postoperative mortality and survival

**Fig. 4:**
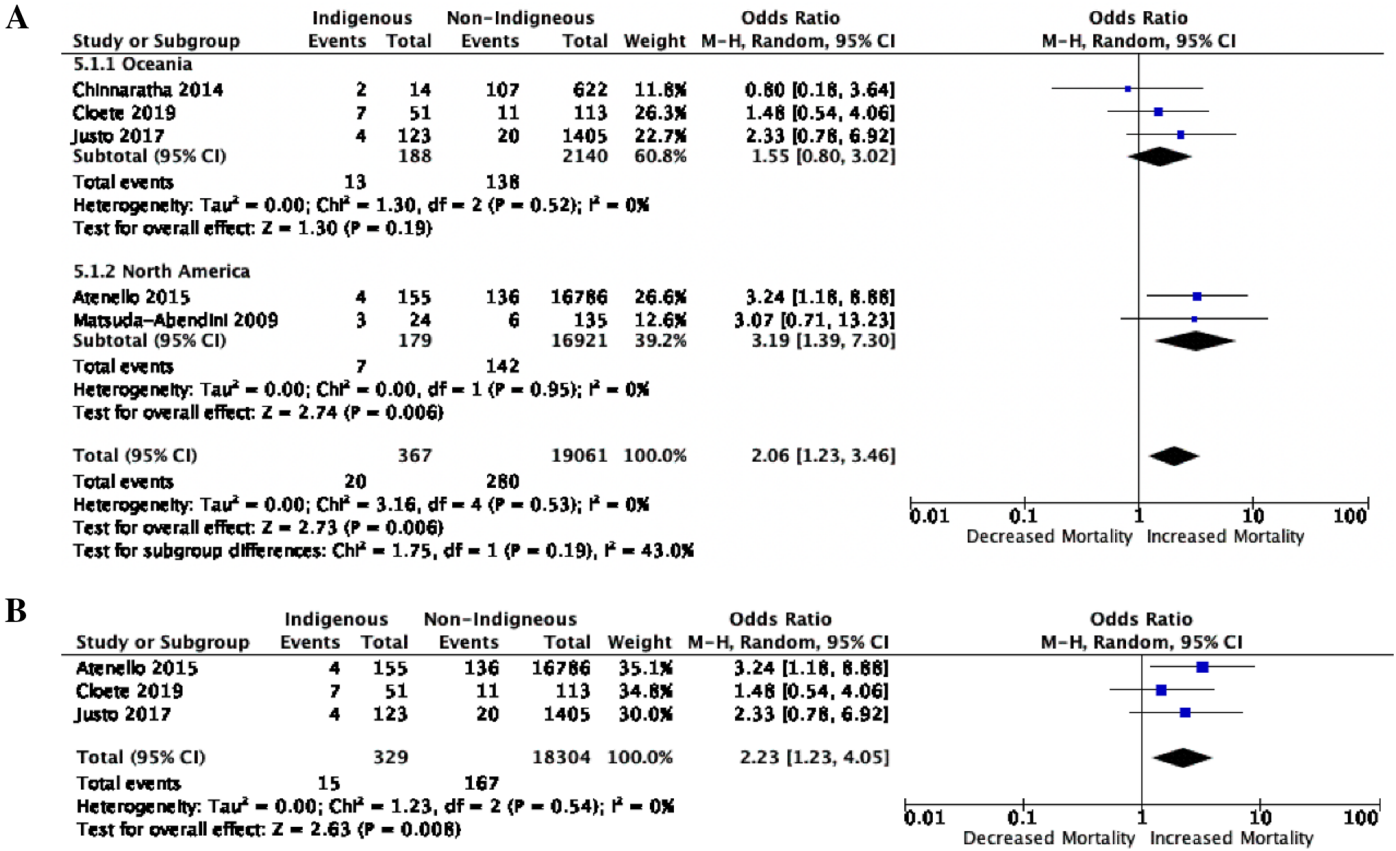
**A** Overall mortality with geographic-specific sub-group analysis and **B** in-hospital and 30-day postoperative mortality rates between Indigenous and non-Indigenous pediatric patients

Six studies addressed postoperative mortality, of which five studies were included in the mortality meta-analysis, reflecting 367 Indigenous and 19,061 non-Indigenous postoperative deaths [19, 21, 25, 27, 30]. The excluded study did not present appropriate data for inclusion in this meta-analysis (HR only) [23]. Three studies provided data on in-hospital and 30-day mortality [21, 27, 30] and two provided data for greater than 30-day mortality [19, 25]. Overall mortality was significantly higher for Indigenous patients compared to non-Indigenous patients (OR 2.06, 95% CI 1.23–3.46, *p* = 0.006; Fig. 4A). Similarly, 30-day mortality was significantly increased for Indigenous patients compared to non-Indigenous patients (OR 2.23, 95% CI 1.23–4.05, *p* = 0.008; Fig. 4B). When stratified by geographic location, Indigenous children in North America had greater than 300 × the odds of postoperative mortality (OR 3.19, CI 1.39–7.30, *p* = 0.006; Fig. 4A). In contrast, Indigenous children from Oceania did not have a significant increase in surgical mortality compared to non-Indigenous patients (OR 1.55, 95% CI 0.80–43.02, *p* = 0.19; Fig. 4A. In a study that examined 5-year surgical survival, Indigenous children in Canada had 50% lower survival than their non-Indigenous counterparts following renal transplantation (*n* = 3, *p* = 0.03) [19]. Deaths in the Indigenous patients were attributed to a cardiac event (*n* = 1), respiratory failure (*n* = 1), and unknown cause (*n* = 1). In the non-Indigenous group, the causes of death were due to a cardiac event (*n* = 1), malignancy (*n* = 1), CVA (*n* = 1), and unknown causes (*n* = 3). Conversely, in a similar study comparing post-liver transplantation survival for Aboriginal and Torres Strait Islander children to non-Indigenous Australians, there was no significant difference in 1- to 15-year survival between the two groups [25].

### Healthcare system interactions

**Fig. 5:**
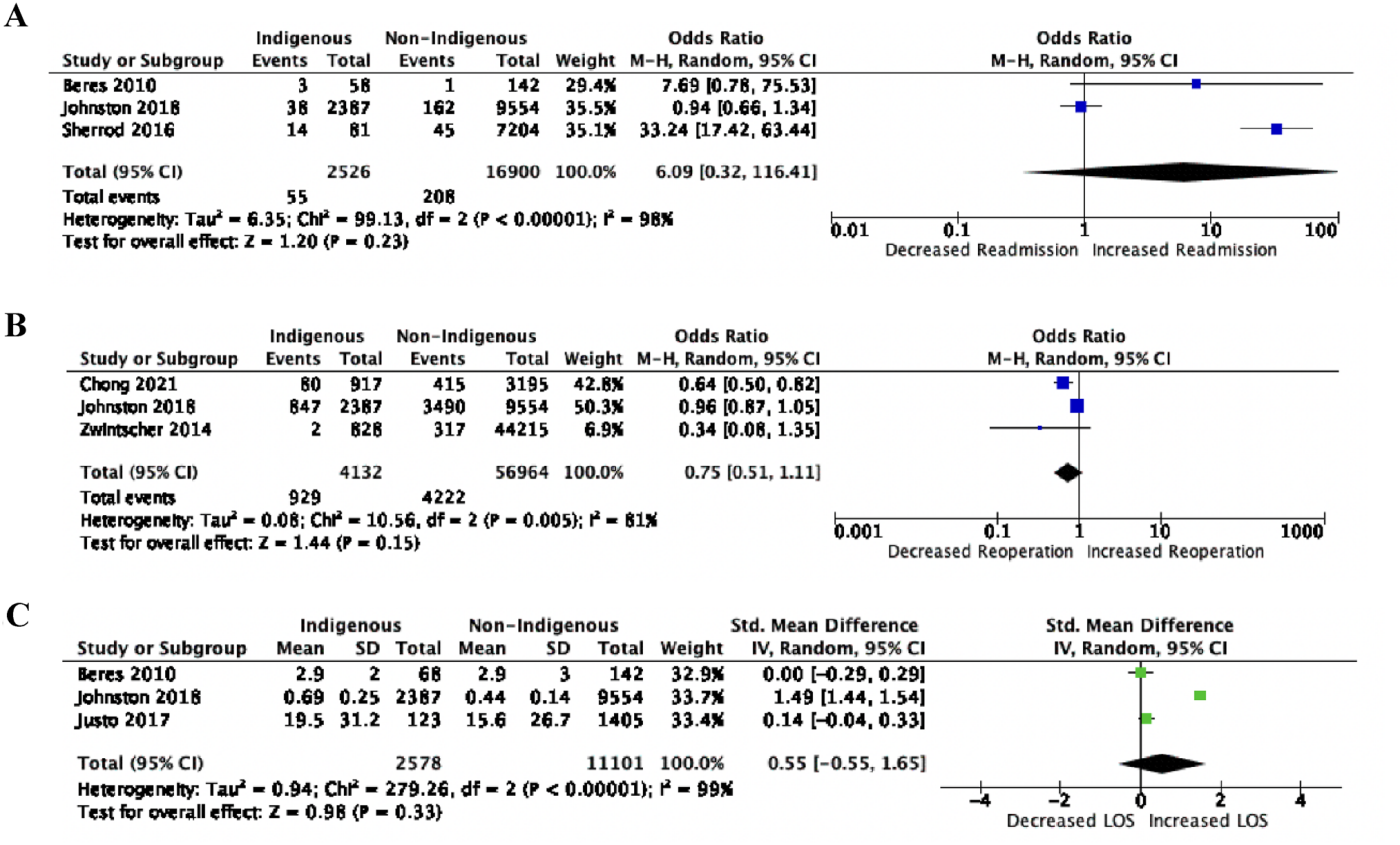
**A** Postoperative readmission rates **B** reoperation rates and **C** length of hospital stay (days) differences between Indigenous and non-Indigenous pediatric patients

Six studies were included in the meta-analysis on reoperation rates, readmission rates, and average length of hospital stay [18, 22, 24, 27, 29, 31]. Indigenous patients had a non-significant increase in hospital readmissions post-operatively (OR 6.09, 95% CI 0.32–116.41, *p* = 0.23) and an increased average hospital LOS (SMD = 0.55, 95% CI − 0.55–1.65, *p* = 0.33) (Fig. 5A, 5C). There was no significant difference in reoperation rates between Indigenous and non-Indigenous patients (OR 0.75, 95% CI 0.51–1.11, *p* = 0.15) (Fig. 5B). Of note, one of the studies reporting on reoperation rates noted that Māori and Pacific Islander children received significantly less reoperations following failed strabismus repairs compared to non-Indigenous children [29].

### Sensitivity and sub-group analyses

We performed a sensitivity analysis to test the assumption that a random-effects method was the most appropriate choice for the analysis. No noticeable change in the direction of the effect with a fixed-effects method was appreciated. A secondary sensitivity analysis was planned based on quality of studies; however, as only one study was good quality, this analysis was not possible. There were no enough studies on specific surgeries to conduct a surgical procedure-specific sub-group analysis.

## Discussion

To the best of the authors’ knowledge, this scoping review and meta-analysis is the first to comprehensively analyze postoperative outcomes in pediatric Indigenous populations in the Americas and Oceania. Our work showed that Indigenous children have a greater than twofold increase in 30-day and long-term postoperative mortality compared to non-Indigenous children. Additionally, while overall postoperative morbidity rates were not found to be significantly different, several studies indicated that Indigenous patients face greater postoperative morbidity than non-Indigenous patients, including increases in hospital readmission rates, decreased rates of reoperations for failed surgeries, and lower rates of graft survival following renal transplantation. Thus, we present evidence that Indigenous pediatric patients suffer from inequitable postoperative outcomes worldwide. The evidence presented here strengthens findings in the literature that indicate the dire need to remodel current surgical systems in a manner that directly reduces inequities and barriers for Indigenous children in accessing safe and effective surgical care [13, 32].

While the reasons as to why increased postoperative morbidity and mortality exist among pediatric Indigenous versus non-Indigenous populations is out of the scope of this review, several important determinants are worthy of note. Poorer health outcomes experienced by Indigenous peoples are often attributed in large part to the rurality and remoteness of areas in which many Indigenous populations live, based on the assumption that this delays access to surgical care and increases late-stage disease presentation. However, an Australian study from 2016 that stratified Indigenous versus non-Indigenous patients by rurality status demonstrated that both rural and urban Indigenous patients experienced significantly higher rates of adverse post-renal transplantation outcomes compared to non-Indigenous rural and urban populations [33]. Similarly, other studies that control for rurality when assessing postoperative mortality and morbidity between Indigenous and non-Indigenous patients in North America and Oceania also conclude that Indigenous status alone, not distance from urban centers, portends worse surgical outcomes [34, 35, 36]. However, when rurality and remoteness do affect health outcomes, it is important to acknowledge that until the past few decades, governmental policies were created and enforced to purposely exclude Indigenous peoples from urban centers, which likewise continues to influence the lives of rural and remote-dwelling Indigenous peoples today [3].

Factors other than rurality and remoteness must also be explored as they contribute significantly to the discrepancy in surgical outcomes for Indigenous children. In their hallmark review, McVicar et al. describe how potential contributors to increased mortality for Canadian Indigenous populations may include late-stage disease at presentation, national surgical referral patterns, and inadequate systems for transition to follow-up care [32]. Undeniably, the ongoing effects of colonization and systemic racism continue to greatly affect the health of pediatric Indigenous populations worldwide. These inequities, driven by culturally insensitive healthcare systems, care teams, and socioeconomic factors [3, 7], must be recognized for their potential to augment traumatic morbidity and increase risk of death following surgery in children. To truly improve a surgical system, it is essential to continue to explore, address, and tackle these complex and intertwining factors.

In addition to describing the increased postoperative mortality and morbidity for Indigenous children globally, this study also highlights the lack of high-quality studies on surgical outcomes in Indigenous children, including the paucity of studies in Canada and the complete absence of data from Central and South America. This is particularly disappointing given the fact that both North and South America have high gaps in life expectancy for their Indigenous populations, with Indigenous Peoples having up to a 16-year less life-expectancy than non-Indigenous people [37]. When adjusted for geographic location, only the North American studies continued to demonstrate significantly increased post-surgical mortality for Indigenous children, indicating differences in postoperative mortality among the diverse Indigenous populations across America and Oceania.

In recent years, scrutiny and increased attention has been directed at the inequitable social determinants of health faced by Indigenous Peoples worldwide, which has resulted in efforts to address these inequities such as the implementation of cultural safety training programs and the development of focused political and health policy agendas [38, 39]. This review is inclusive to the end of 2021; however, the most recent cohort year included in the meta-analysis is 2014. While concrete evidence of positive change is yet to be seen, as further research emerges on this topic, it will be interesting to explore if policy and cultural changes in the last decade have created meaningful improvements in the health of Indigenous children.

The results of this scoping review are consistent with the United Nations Mandate on Indigenous Peoples Health which describes how Indigenous Peoples across the globe face overall poorer health outcomes, reduced quality of life, and higher rates of disability compared to non-Indigenous peoples [37]. Ultimately, we present the disheartening but critically important finding that these inequities in health outcomes begin in infancy and childhood, manifesting as steeply elevated postoperative mortality rates and increased risk of complications following surgery for Indigenous children.

Based on this study’s findings, we propose the need for further research and health systems reforms that are appropriate for the Indigenous context and enshrine in their models the need for multi-pronged solutions that respect the unique cultures, experiences, and needs of diverse Indigenous communities worldwide. Further research is required to investigate rates of and structural factors influencing inequities in postoperative morbidity and mortality in Indigenous pediatric populations, especially in the Americas. The pediatric context must be recognized as unique and treated accordingly in both research and practice to ensure their right to equitable and safe healthcare is upheld. These research initiatives should involve a clear methodology that is developed in collaboration with Indigenous leaders, communities, and healers, with specific care taken to incorporate and accommodate all of the diverse Indigenous groups of a given region. End goals must emphasize the transformation of research into practice by reforming various steps in the surgical care pathway in manners that radically improve access, safety, cultural-appropriateness, and outcomes for Indigenous children.

### Limitations

Heterogeneity present in the research question, including the diversity of Indigenous populations, surgeries performed, surgical procedures, and reported outcomes, makes it difficult to pool the results for this meta-analysis in a manner that reflects nuances between the study populations, while also providing homogeneity in pooled odds. The studies included in this review were also primarily of poor quality and retrospective in nature. Furthermore, the majority of studies included in this review did not explicitly define or provide separate outcome numbers for unique Indigenous groups of a specific geographic region, labeling their populations strictly as “Indigenous” and grouping together several geographically, culturally, and ethnically diverse Indigenous groups. This homogenization of Indigenous groups consequently does not allow for analysis of unique experiences and barriers for these distinct groups.

It is important to acknowledge that inequities in health outcomes between Indigenous and non-Indigenous populations impact patients at many stages of their treatment journey. While we compare inequity in surgical outcomes, the impact of contextualizing inequities leading up to surgery is outside the scope of this review. Questions such as: “Are Indigenous and non-Indigenous patients presenting with similar clinical pictures equally likely to receive appropriate surgical intervention?” and “Are negative surgical outcomes reported at equal rates in Indigenous and non-Indigenous populations?” remain. Furthermore, while this review did not find any studies from South or Central America, there is a possibility that this may have been influenced by our search strategy being limited to the English language.

### Supplementary Information

Below is the link to the electronic supplementary material.

Supplementary file1 (DOCX 14 kb)

## Data Availability

The datasets generated and/or analysed during the current study are available from the corresponding author on reasonable request.

## Abbreviations

ACE: Adverse childhood experiences
CVA: Cerebrovascular accident
ENT: Ears, nose, and throat surgery
GS: General surgery
ICU: Intensive care unit
LOS: Length of stay
M&M: Morbidity and mortality
MI: Myocardial infarction
MOOSE: Meta-analysis of observational studies in epidemiology
NOS: Newcastle–Ottawa scale
NS: Neurosurgery
ORTHO: Orthopedic surgery
PRISMA-ScR: Preferred reporting items for systematic review and meta-analysis-extension for scoping reviews
RoB: Risk of bias
SMD: Standard mean difference
SSI: Surgical site infection
QA: Quality assessment
URO: Urology
USA: United States of America
