# Supplementary material for: Postoperative morbidity and mortality in pediatric indigenous populations: a scoping review and meta-analysis
Source: Pediatr Surg Int. 2023 Feb 16;39(1):129. doi: 10.1007/s00383-023-05377-2 (PMC9935719; doi:10.1007/s00383-023-05377-2)
Supplement: Supplementary file 1 — Supplementary file1 (DOCX 14 kb) [file 383_2023_5377_MOESM1_ESM.docx]

Supplementary File 3. Summary of search strategy.

| **Source** | **Database(s)** | **Search Terms Example** | **Inclusion Criteria** | **Exclusion Criteria** |
| --- | --- | --- | --- | --- |
| Indexed Peer-Reviewed Journals | MEDLINE, Embase, Global Health, Cochrane Library, PsycInfo, SOCIndex, Web of Science, ProQuest Dissertations & Theses Global | ((ti((aboriginal OR "first nation" OR "first nations" OR inuit OR metis OR "indigenous canadians" OR "native people" OR "native peoples" OR "on-reserve" OR "off-reserve")) OR ab((aboriginal OR "firstnation" OR "first nations" OR inuit OR Alaskan native OR Maori OR metis OR "indigenous canadians" OR "native people" OR "native peoples" OR "on-reserve" OR "off-reserve"))) OR noft((Algonquin OR Aleut* OR Anishinabek OR Anishnabek OR Chipewyan OR Cree OR Dene OR eskimo* OR Gitskan OR Huron OR Innu OR Inuktitut OR Maori OR Amerid* OR "Indians, South America*" OR Quechua OR "Pacific Islander*" OR Chiquitano OR Mesitzo OR Guaranie* OR Inuk OR Inupiat* OR Iqaluit OR Iroquois OR Kalaallit* OR "Kawawachikamach Québec" OR Kahnawa:ke OR Kitikmeot OR Kitimat OR Kivalliq OR Kwakiutl OR Manitoulin OR Metis OR Miawpukek OR Micmac OR Mi’kmaq OR Mohawk OR Mushkegowuk OR Naskapi OR Nisga'a OR Nakoda OR Nakota OR Oji-Cree OR Ojibway OR Oki OR Opaskwayak OR Pauktuutit OR Qikiqtani OR Qayuqtuvik OR "Rankin Inlet" OR Sekon OR Sioux OR Tungasugit OR Tuttarvingat OR "Vuntut Gwitchin"))) AND (ti(surger* OR surgical OR operation OR operative OR transplant*) OR ti(obstetric* OR cesarean OR caesarean OR c-section) OR (ab(perioperative* OR peri-operative) OR ab(preoperative* OR pre-operative*) OR ab(intraoperative* OR intra-operative*) OR ab(postoperative* OR post-operative* OR postsurg* OR post-surg*)) OR su((surger* OR surgical OR operation OR operative OR transplant*))) | (a) Experimental or observational studies  (b) Indigenous population in NA, SA, or Oceania  (c) Non-Indigenous comparator group  (d) Surgical intervention  (e) Post-operative outcomes (morbidity and/or mortality) reported  (f) English language | (a) Book chapters, conference abstracts, and/or non-peer reviewed articles  (b) Focused on Indigenous populations outside of NA, SA, or Oceania  (c) Lacked non-Indigenous comparator group  (d) Intervention non-surgical (including minor interventions and procedures conducted by interventional medical specialists including but not limited to: angiography, bronchoscopy, colonoscopy, gastroscopy, bone marrow biopsies, and percutaneous procedures)  (e) Pre-operative or intraoperative outcomes only  (f) Non-English language |
| Other: Grey literature, reference lists of reviews and retrieved articles; consultation with experts | N/A |  |  |  |
